# New Appliances and Things Medical

**Published:** 1896-02-29

**Authors:** 


### NEW APPLIANCES AND THINGS MEDICAL.

[We shall be glad to receive, at our Office, 428, Strand, London, W.C., from the manufacturers, specimens of all new preparations and appliances, which may be brought out from time to time.]

### THE MAKING OF VINOLIA.

(Blondeau et Cie., Rylands Road, N.)

Curiosity lately tempted us to make a pilgrimage into the North of London to see the extensive factory where Messrs. Blondeau make Vinolia, meaning thereby not the well-known soap alone, but the powder, the cream, and the various scents and toilet requisites with which they supply a grateful world in such abundance. It is a large concern, occupying an extensive range of well-built factory premises, and even this large building does not include all, for the boiling of the soap is done elsewhere. The soap then arrives at the factory already made in the form of bars of various qualities, and it is with the testing, drying, and mixing of these and their scenting and formation into tablets that this great factory is occupied. First the bars are sliced; then the slices are ranged on shelves in rooms through which is driven a vast quantity of warm dry air, by which the moisture is gradually carried off; after this the slices are broken up and mixed in the proportions necessary to make the various qualities. These mixtures, each in a machine of its own, are then compressed, and made to issue in the form of a thin white shaving, clean and delicate in appearance. This process serves several purposes—it still further dries the soap, it mixes it even more thoroughly than before, and it enables the scent, when it is applied, to permeate every portion of the mass. When the appropriate scent has been added these shavings are again compressed into solid bars, out of which, as they issue from the machine, the future tablets are roughly stamped, much as a cook would cut rings of pastry for making tartlets. Each of these is then picked up by hand and placed in a powerful press furnished with gun-metal dies, by which it is stamped into its final form, and obtains the impress of its well-known name. Each tablet as it comes from the die is placed on a tray as delicately as the pastrycook would place his tarts, and sent upstairs, where, after any little roughness at the edges has been removed, it is enfolded in its final wrapper of variously-decorated paper. The whole process is one of great nicety and cleanliness. The men and boys engaged in the factory and the long rows of tidily-dressed girls by whom the soap was being trimmed and wrapped up looked healthy, and seemed to be of a class somewhat superior to mere factory workers. Not the least interesting place included in our visit was the laboratory, where two qualified chemists are constantly engaged in analysing the soap in its various stages, and in carefully testing and examining, spectroscopically and otherwise, the large quantities of attar of roses and various perfumes which are used in the scenting of the soap, in the manufacture of perfumery, and for other purposes. The wily Roumanian who grows the roses is by no means above a little sophistication of his wares, and if he can get a little geranium essence accepted as attar of roses he is very glad, and it is to the careful testing of everything they use that Messrs. Blondeau attribute no small share of their success. Much more we saw which we need not here describe, but we came away well pleased with the cleanly surroundings amid which all the processes of soap manufacture which we saw were carried out.

### REFINED BEEF SUET "ATORA" BRAND.

(Hugon and Co., Manchester.)

This article is simply beef suet, rendered and refined by a special process which should ensure it keeping fresh and sweet for an almost indefinite time, if kept in a cool and dry place. Hugon's refined beef suet is guaranteed by the makers to be rendered from fresh English beef suet only. In the refining process the suet is freed from all tissue and moisture, so that the material represents a pure animal fat containing no impurities. We have submitted a sample of this suet to our analyst, who reports that he found it to contain less than 0·18 per cent. of water, and that it left practically no ash on ignition. An examination of the fat showed that it consisted of genuine ox fat, without any admixture with cotton seed oil or other foreign fats, and the sample examined was neutral, and free from any signs of decomposition. We have not tested its keeping power, but as it is put on the market in solid blocks there is every reason for believing that a fat of this purity would remain sweet for a very long time, and as portions can be shedded off as required we are of the opinion that it would be found most economical for general household purposes, and recommend a trial to those engaged in the culinary art.

